# Surgical excision of Essure® devices with ESHRE Class IIb uterine malformation: sequential hysteroscopic-laparoscopic approach to the septate uterus

**Published:** 2016-03-28

**Authors:** ES Sills, GD Palermo

**Affiliations:** Reproductive Research Division, Center for Advanced Genetics; Carlsbad, California, USA.; Molecular and Applied Biosciences Department, Faculty of Science & Technology, University of Westminster, London, UK.; Ronald O. Perelman & Claudia Cohen Center for Reproductive Medicine, Weill Cornell Medical College, New York, USA.

**Keywords:** Essure, contraception, reproductive surgery, ESHRE class IIb uterine malformation

## Abstract

**Objective:**

While contraindications to Essure® placement have been provided by the manufacturer, there is no consensus on how best to remove these contraceptive devices. Here, we describe a non-hysterectomy removal of Essure® for a patient with a septate uterus (ESHRE Class IIb uterine malformation).

**Clinical case:**

A 35yr old G4 P2 presented for removal of Essure® implants after three years of gradually increasing pelvic pain, weight gain, headache, dizziness, lower extremity paresthesia, and fatigue which followed hysteroscopic sterilization (HS). Prior to HS, the patient was in good general health. She did not smoke and had never had a miscarriage. HS was performed under general anesthesia in October 2012. HSG obtained three months later, confirmed bilateral tubal occlusion but revealed an abnormal uterine cavity. A repeat HSG in 2015 showed minimal device migration, no contrast dye spill and a deeply bifid uterine cavity. At our center laparoscopic cornual dissection and bilateral partial tubal resection achieved removal of both devices intact and the patient was discharged three hours after surgery. Her postoperative recovery was uneventful.

**Conclusion:**

The presence of a Müllerian anomaly is a relative contraindication to the Essure® procedure. This is the first reported description of successful removal of Essure® coils in the setting of an ESHRE Class IIb uterine anomaly, and underscores the importance of careful patient selection, accurate pre-operative imaging and a conservative technique which renders hysterectomy unnecessary.

## Introduction

Most research on uterine anomalies has derived from populations of women with a history of reproductive loss, so there is little data on how prevalent this condition may be in the general population. One investigation, which screened asymptomatic women with no adverse reproductive history, reported that approximately 3% had a septate uterus (Woelfer et al., 2001). Among infertility patients however, this condition is thought to be present in up to 10% of women (Propst and Hill, 2000). One prospective analysis from the United Kingdom showed that women with a septate or bicornuate uterus experienced significantly increased second-trimester miscarriages compared to patients with no uterine malformation (Saravelos et al., 2010).

Each year, more than 300,000 women request permanent surgical sterilization in the United States (Jones et al., 2012), and it is possible that some of these patients will have an undiagnosed Müllerian anomaly. The current classification system of uterine malformations uses a taxonomy developed from an international consensus conference (Grimbizis et al., 2016). While laparoscopic bilateral tubal ligation is the most common technique to provide permanent female contraception, a new sterilization option became available in 2002: bilateral tubal occlusion via hysteroscopic insertion of nickel-titanium inserts at the proximal fallopian tubes. Importantly, the presence of a uterine malformation may impair visualization of tubal ostia and thus is listed as a relative contraindication for the Essure® procedure. Nevertheless, the Essure® procedure can be performed in the setting of an ESHRE Class IIb uterine malformation although no data exist to describe how often this occurs. The current report is the first to describe the Essure® technique applied to a woman with a uterine septum and the successful removal of these devices using a minimally-invasive, uterus-sparing surgical approach.

## Clinical Case

A 35-year-old G4P2 attended for reproductive surgery consultation to discuss a variety of problems she began to experience subsequent to undergoing the Essure® procedure for permanent sterilization. HS was performed in October 2012 under general anesthesia; pre-procedure pelvic ultrasound was unremarkable. Before HS, the patient was in good general health and all of her pregnancies were established without medical assistance. Cervical cytology was routinely normal, the patient did not smoke and she never had a miscarriage. Soon after the Essure® devices had been placed, the patient reported gradually increasing pelvic pain, headache, dizziness, lower extremity paresthesia and fatigue. In the first six months following the Essure® procedure, the patient registered a 20 pound weight gain. The patient’s concerns persisted over a three-year interval, although her gynecologist was disinclined to attribute any of the problems to Essure®.

Three months after HS, the patient presented for hysterosalpingogram as recommended. While the study did confirm bilateral tubal occlusion, it also revealed an abnormal (bifid) uterine cavity. 3-D ultrasound or other imaging was not performed to clarify or refine the diagnosis of the uterine anomaly and the patient received no further counseling.

**Fig. 1:**
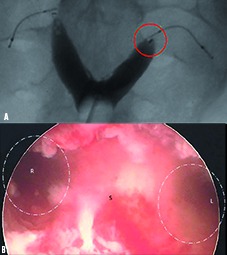
— Preoperative HSG (a) showing bifid uterine cavity, Essure® devices and bilateral tubal occlusion. The implant on the left appears to extend into the uterine compartment (circle). Hysteroscopic view (b) demonstrating essentially symmetric uterine division by the septum (S).

The patient later requested surgical removal of the Essure® implants and the possibility of device migration required evaluation (Ricci et al., 2014). A repeat HSG was obtained in 2015. This study revealed minimal device migration and no contrast dye spill from either Fallopian tube, a deeply bifid uterine cavity was again the most conspicuous finding (Fig. 1a).

**Fig. 2:**
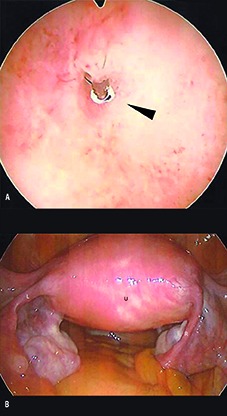
— Hysteroscopic view of left tubal ostia (a) showing minimal protrusion of Essure® device (arrow). Exterior uterine contours at laparoscopy (b) were consistent with uterine septum (ESHRE Class IIb malformation).

At our center the patient was counseled about the atypical nature of her Essure® placement given the anatomical abnormality of the uterus. After obtaining informed consent, the patient underwent diagnostic hysteroscopy which verified an abnormal bifid uterus (Fig. 1b). The ostia on the right was normal as was the entire right uterine segment. However, the Essure® device on the left partially extended into the endometrial compartment (Fig. 2a). The exterior uterus was consistent with ESHRE Class IIb anomaly (uterine septum) (Fig. 2b). There was no evidence of tissue perforation by either contraceptive device. Laparoscopic dissection of the left cornu and partial tubal resection bilaterally achieved complete removal of the Essure® implants. Removal of terminal markers was confirmed. Electrocautery was maintained at 40 W power throughout the case, blood loss was estimated to be less than 50 mL. The patient was discharged three hours after surgery and her postoperative convalescence has been uneventful.

## Discussion

The reproductive history of most women with a Müllerian malformation is generally poor, especially for patients with a uterine septum, which is the most common anomaly (Propst and Hill, 2000). This report describes a case which is exceptional in two respects – a patient with no adverse reproductive history despite having a uterine septum, and who also underwent hysteroscopic sterilization with Essure®. As the presence of an abnormal uterine cavity is a relative contraindication to the Essure® procedure, it is unclear why this individual was selected for hysteroscopic sterilization. In any case, she did develop a number of symptoms shortly after receiving the Essure® implants and sought surgical excision of these devices while sparing her uterus. Of note, it was during the pre-operative evaluation that the patient became aware of her uterine cavity defect and only at surgery the definitive diagnosis was made. Both contraceptive devices were successfully removed with excision of terminal markers using a previously described technique (Sills et al., 2015).

Only 20 reports on Essure® removal were published to date, although ours is the first known description of excision of Essure® devices in the setting of an ESHRE Class IIb uterine malformation. The fact that our patient required additional surgery after her Essure® procedure does align with recent research on hysteroscopic sterilization which found a substantially elevated risk of reoperation among these women compared to those who elected conventional laparoscopic sterilization (Mao et al., 2015). Our patient’s post-operative recovery was unremarkable, her symptoms associated with Essure® have largely resolved.

The Essure® sterilization technique occupies a rather unusual domain within the terrain of contraceptive choices and the cumulative published experience with the device remains surprisingly limited considering how long the product has been clinically available. There is even disagreement regarding how best to describe the procedure: Should Essure® be classified as "non-surgical" or "non-incisional"? Given the sometimes protean complaints which can occur after HS, the potential for autoimmune inflammatory syndrome induced by adjuvants (ASIA syndrome) may warrant consideration (Vera-Lastra et al., 2013). Our patient’s story also highlights the problem of uneven familiarity among physicians regarding patient selection for hysteroscopic sterilization. Therefore counseling patients seeking advice on contraceptive choices is frustrated by the limited literature addressing the overall epidemiology of Essure®. These factors underscore the immediate need to increase awareness of the failures – and successes – of HS. We believe that assignment of unique ICD-10 modifiers for Essure® complaints would afford clinicians and health policy researchers an important tool to monitor the Essure® phenomenon. While ICD-10 codes exist for unusual clinical events like "sucked into jet engine, subsequent encounter" (V97.33XD) and "other contact with cow" (W55.29XA), there unfortunately remains no specific diagnostic coding available for Essure® associated symptoms (Williams, 2015). Further research regarding hysteroscopic sterilisation is needed as this contraceptive option becomes more widely accessed.
